# Effects of Baduanjin on patients with chronic nonspecific low back pain: A randomized controlled trial

**DOI:** 10.1097/MD.0000000000024448

**Published:** 2021-01-29

**Authors:** Qingtang Yang, Shiliang Yu, Jianbin Wang, Caiyun Zheng, Xiaofeng Liang, Debiao Yu, Xiangmei Chen

**Affiliations:** a Department of Rehabilitation, First Hospital of Nanping City; b Department of Rehabilitation, Nanping People's Hospital, Fujian Province, Nanping; c Department of Rehabilitation, Fujian Provincial Hospital, Fuzhou, Fujian, China.

**Keywords:** Baduanjin exercise, chronic low back pain, clinical efficacy, tandomized controlled trial protocol

## Abstract

**Background:**

Chronic low back pain (CLBP) is 1 of the common clinical diseases, and many treatment methods can only improve the symptoms of pain in the short term. Traditional Chinese sports - Baduanjin has been proven to have a positive effect on chronic low back pain. However, the quality of the research is low, the sample size is small, and safety observations are lacking. We describe the protocol of a randomized controlled trial to study the efficacy and safety of Baduanjin chronic low back pain.

**Methods:**

This randomized, controlled, evaluator-blind, two-arm, parallel clinical trial will include 90 outpatients with chronic low back pain recruited from the First Hospital of Nanping City, Fujian Province. The patients were randomly assigned to the intervention group (Baduanjin exercise training) and the control group (not receiving any special exercise training) at a ratio of 1:1. Patients in the intervention group will receive Baduanjin exercise training 3 times a week for 24 weeks. The 2 groups received a 4- week follow-up observation at 24 weeks. The main result from the intervention before intervention to 24 weeks later, and the follow-up of 4 changes the visual analog scale score at weeks, and by independent t are tested groups. It will also review the Pain-related disability index, The Quebec Back Pain Disability Scale, Health-related quality of life, Roland Morris (Roland Morris) Disability Questionnaire, Overall Perceived Effect (OPE) and safety Compare. Cost data for cost-benefit and cost-benefit analysis will be collected.

**Discussion:**

This will be the first study to compare the effectiveness and safety of Baduanjin for patients with chronic low back pain. The results may help healthcare professionals make clinical decisions and may reduce the cost of treatment for this disease.

**Trial registration:**

ChiCTR2000033908

## 1 Introduction

With the changes in human living environment and working conditions, Low Back Pain has become 1 of the most common public health problems in the world.^[1]^ Epidemiological surveys show that the point prevalence of low back pain is about 18%, and the lifetime prevalence is about 40%.^[2]^ Although many people's low back pain will recover within a year, more than 5% to 10% of patients will have persistent low back pain.^[3,4]^ When this kind of low back pain lasts for more than 3 months, it develops into a disease---Chronic Low Back Pain (CLBP).^[1,5]^ The dysfunction of low back pain caused by CLBP has different degrees of negative impact on patients’ mobility, daily function, work ability and quality of life,^[6–8]^ and is also the most common cause of disability among people aged 45 to 85.^[3,9]^ At the same time, it causes great burden to the patient's individual, family and social economy.^[10]^ Since the vast majority of low back pain patients are non-specific pain, the underlying pathological or harmful factors have not yet been identified.^[11]^ Although the evaluation and treatment methods have improved, the management of low back pain is still a challenge for researchers and clinicians.

Many clinical practice guidelines recommend various methods to evaluate and manage CLBP.^[12–14]^ These guidelines all mention the prudent use of imaging evaluations, pain medications, spinal injections, and surgical procedures.^[15]^ Unfortunately, these therapies are not always effective and may increase some adverse reactions, including nausea, vomiting, insomnia, headaches and disability. Other recommended management methods, including self-management, restorative activities and exercise, are considered effective methods.^[14,15]^ However, a unified management model has not yet been formed,^[16]^ and there are large differences in practice in different countries and regions. Although exercise has been recognized as a moderately effective method for the treatment of CLBP,^[17,18]^ there is limited data on the cost-effectiveness of some exercise treatments, which require expensive specialized equipment.

Baduanjin is a traditional Chinese way of exercise, which consists of 8n simple, independent and smooth movements.^[19]^ Although the results of each movement may be different, in general Baduanjin can promote the coordination of mind and body, thus regulating respiration and playing an important role in health care.^[19,20]^ In recent years, Chinese medicine has received more and more attention from scientific research circles, especially its benefits to human body and mental health.^[21]^ Baduanjin has been proven to be safe and effective,^[22]^ it can improve the physical condition of patients, such as reducing the symptoms of Low Back Pain, enhancing physical function, and reducing self- reported disability.^[23]^ In general, it can actively adjust various psychological indicators, such as anxiety, irritability, depression and concentration.^[24]^ In China, Baduanjin is a commonly used and widely used treatment method to relieve the symptoms of lower back pain, with relatively remarkable effect.^[23]^ It mainly through strengthening the back chest abdominal muscles and bones, adjust the cervical spine thoracic lumbar physiological curvature, to reduce the pain of patients.^[25–27]^ At present, the effectiveness of Baduanjin in the intervention of CLBP has been confirmed by previous randomized controlled trials,^[23,28]^ but these studies have some problems, such as small sample size, simple observation indicators and lack of safety observation.

Here, we describe the protocol of a randomized controlled clinical trial to evaluate the efficacy of Baduanjin intervention for CLBP. We plan to analyze the effectiveness and safety of Baduanjin interventions in terms of pain intensity, disability level, quality of life, and overall impression change through a 24-week exercise program to provide health practitioners with a low-cost and safe way to address this health issue.

## 2 Materials and methods

### 2.1 Objectives

The main objective of this study was to evaluate the effects of Baduanjin on back function in patients with low back pain, including pain intensity, disability level, quality of life, overall impression change, and safety outcomes.

### 2.2 Research design

**Figure 1:**
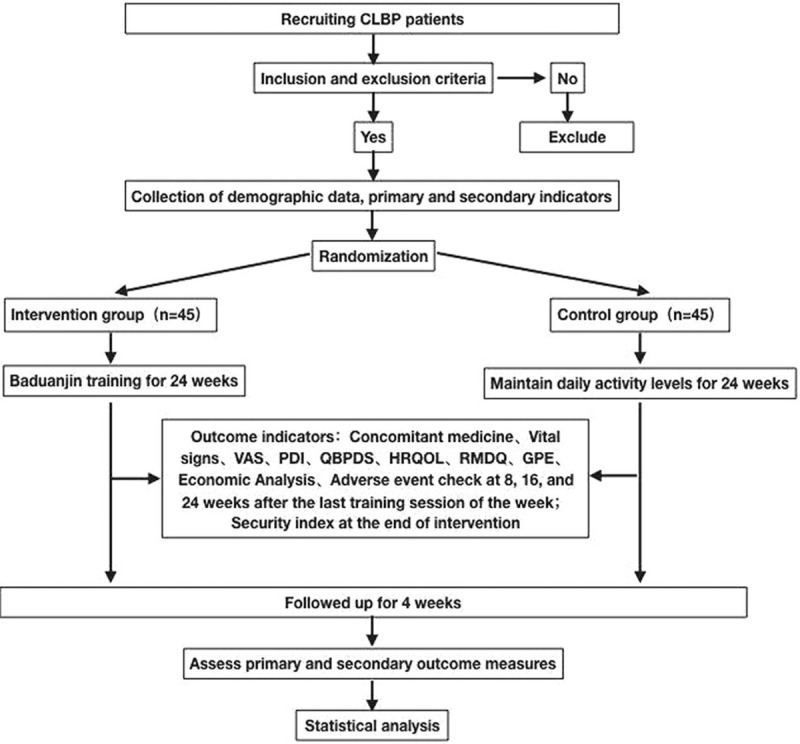
Study design.

This study is a randomized, controlled clinical trials, will conform to the conditions of 90 participants according to the proportion of 1:1 were randomly divided into intervention group (Baduanjin group) and control group (not exercise training group). All the members of the intervention group received Baduanjin training 3 times a week for 24 weeks. All cases in the control group did not receive any exercise training for 24 weeks. Primary and secondary outcomes will be measured at baseline, 8, 16 and 24 weeks after intervention, and the next 4 weeks. The research design flowchart is shown (Fig. 1). The research protocol was described in accordance with “ SPIRIT 2013 statement: defining standard protocol items for clinical trials ”.^[29]^

### 2.3 Inclusion criteria

1. Male and female subjects aged 19 to 70 years;
2. Pain that lasts at least 3 months;
3. At the time of inclusion, pain and discomfort were over 40 mm on the 100 mm Visual Analog Scale (VAS);^[30]^
4. Normal intelligence, able to communicate and communicate, able to complete the basic movements of Baduanjin;
5. Voluntarily provide written informed consent and are willing to follow instructions related to the trial.

### 2.4 Exclusion criteria

1. Suspected or confirmed severe spinal pathology (spinal fracture, metastatic, inflammatory or infectious disease, cauda equina syndrome, diffuse neurological disease);
2. Nerve root injury (the same strength as the nerve root, the effect of sensation or reflex);
3. Past medical procedures, including spinal surgery;
4. The current medical condition plans to undergo major surgery during treatment;
5. Be pregnant, breastfeeding, plan to become pregnant or refuse to use appropriate contraceptive methods during the trial period;
6. Pain in places other than the waist is more severe;
7. Participating in rehabilitation training and exercise therapy influencing the outcome of the trial within the last 2 weeks;
8. It is up to the clinical trial investigator to judge whether the trial is qualified.

### 2.5 Subject withdrawal criteria

1. Violate the inclusion criteria or meet the exclusion criteria;
2. Serious adverse events hindered the continuation of the trial;
3. Withdraw the consent of the subject or legal representative;
4. Do not receive the entire course of treatment continuously;
5. The investigator or subject violates the clinical trial protocol;
6. Use of drugs or treatments that may affect the results of clinical trials without the permission of the researcher;
7. The researcher believes that the progress is inappropriate.

### 2.6 Recruitment

Recruiting patients from the Rehabilitation Department of the First Hospital of Nanping City, Fujian Province seeking treatment for CLBP, the trial began in October 2020 and the last follow-up is expected to be conducted in November 2023.

People who may meet the conditions will be screened first to determine their eligibility based on inclusion and exclusion criteria. Qualified personnel will receive data about the trial, and 2 well-trained research assistants will have an in-depth discussion of the provided information. Participants will be asked to sign a written informed consent form, after which a baseline assessment will be conducted.

Patient and public participation in the trial is currently in the recruitment phase. No patients and the public participated in this test.

### 2.7 Sample size calculation

Perform sample size calculations to determine the appropriate number of participants. The main outcome will be the change of VAS score between the intervention group and the control group from before the intervention to the end of the last intervention. As far as we know, a previous clinical trial evaluated the effects of Baduanjin and conventional rehabilitation intervention for 8 weeks.^[28]^ The change of VAS score after treatment group (8^th^ week) was 2.37 ± 1.7 in the treatment group and 3.70 ± 1.39 in the control group. The following indicators will be considered: α = 0.05, statistical power of 95%, and follow-up loss of up to 20%. We use G∗POWER 3.1.9.7 (http://stats.idre.ucla.edu/other/gpower/) to calculate that the sample size of this study is 45 patients per group. Therefore, a total of 90 participants are required.

### 2.8 Random

After the baseline assessment, eligible participants will be randomly assigned to the intervention group or the control group. The random allocation sequence will be generated using the planning program of the statistical software Statistical Analysis System (SAS V.9.0) (SAS Institute Inc., Cary, NC, USA) and managed by an independent research assistant. Will be responsible for randomly assigning sequences and blind codes. The subject's work result (intervention group or control group) will be replaced with the letter “A” or “B” in the first blind code, and the true value of “A” or “B” will be set in the second blind code. meaning. After locking the database, the independent research assistant will move to the statistician through the participant's group code “A” or “B”, and declare it as the “A” or “B” group after completing all data analysis.

### 2.9 Blinding

In this trial, since the focus of the trial is non-pharmacological intervention, it is impossible to blind the participants and physicians, but use blinding for evaluators and statisticians.

### 2.10 Intervention

If necessary, all participants will continue to receive conventional medical treatment or rehabilitation. Conventional medical treatment or rehabilitation will be based on the Chinese association for the study of pain chronic non-specific low back pain Assessment and Management Consensus (2019).^[31]^ At the same time, all participants will receive the same health education during the intervention period. Participants in the intervention group will receive Baduanjin exercise, while participants in the control group will be told to maintain their usual lifestyle.

### 2.11 Study schedule

**Figure 2:**
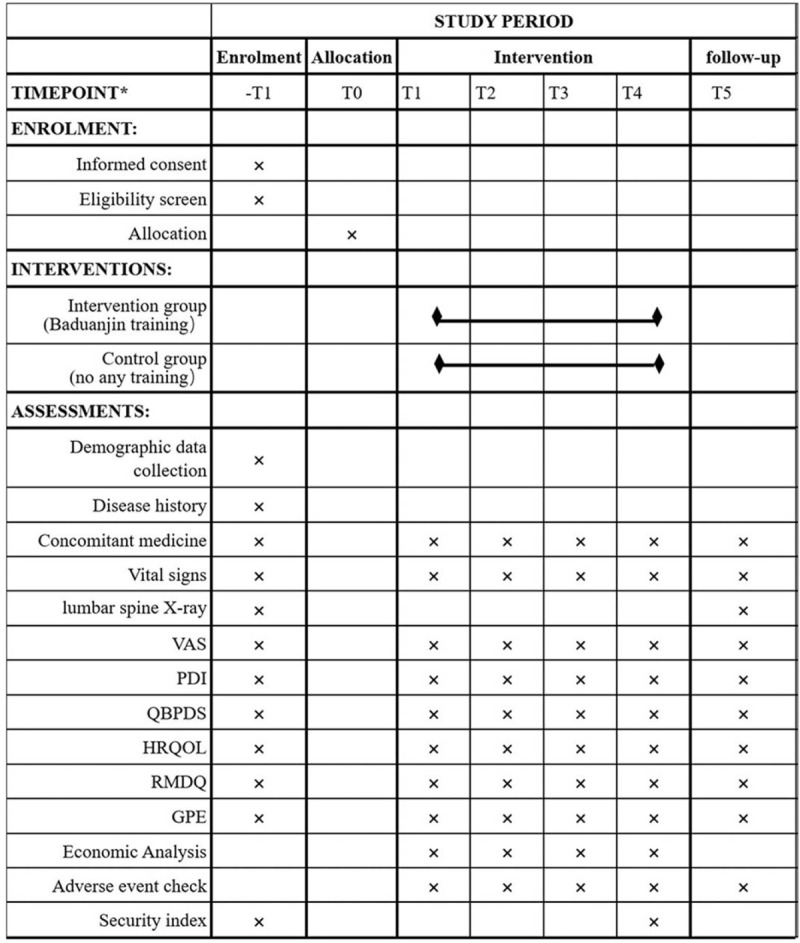
Schedule of enrolment, interventions and assessments. t1 = −2(−1) weeks, 0 = baseline, t1 = week 1, t2 = week 16, t3 = end of treatment week 24, t4 = follow-up week 28.

The research schedule is listed (Fig. 2). The trial will include a screening phase, an intervention phase and a follow-up phase. During the screening phase, participants sign informed consent. Researchers will obtain basic demographic information and use VAS to determine whether participants are eligible for clinical trials. If participants are undergoing rehabilitation training or exercise therapy, we will implement a 2-week flushing period. During the 24- week treatment period, the 2 groups of patients were measured before the intervention, at 8, 16, and 24 weeks after the last training session of the week. In the intervention group, Baduanjin training was performed 3 times a week. The follow-up survey of the intervention group will be conducted 4 weeks after the last training, and the control group will be conducted at the 28th week.

### 2.12 Intervention group

**Figure 3:**
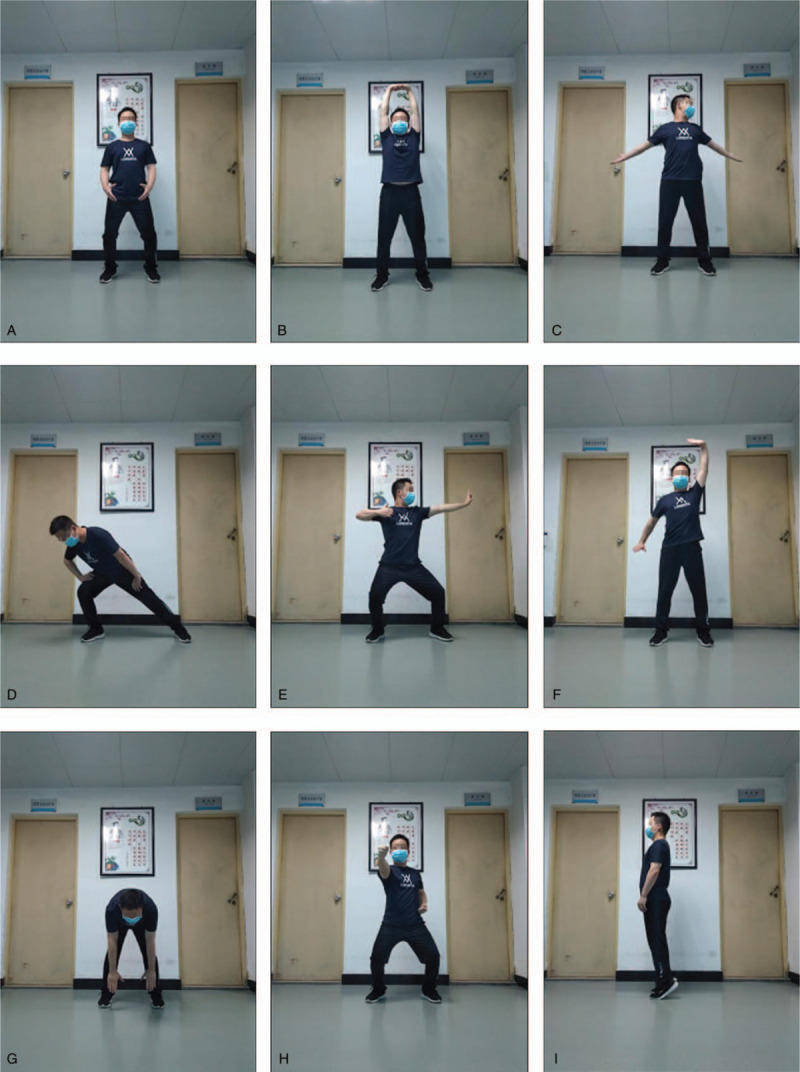
Baduanjin exercise training diagram. (A) Starting form. (B) The first form. (C) The second form. (D) The third form. (E) The fourth form. (F) The fifth form. (G) The sixth form. (H) The seventh form. (I) The eighth form.

Participants in the intervention group will have 24 weeks of Baduanjin exercise training (Fig. 3). At the same time, they will also receive regular medical or rehabilitation treatment and health training. The included patients will receive movement guidance from the teacher of Baduanjin of Fujian university of traditional Chinese medicine before the exercise training of Baduanjin, and begin the exercise training intervention of Baduanjin after they recognize the patient's movement. Baduanjin exercise training will last 24 weeks, 3 days a week, 40 minutes a day. The cultivation plan of Baduanjin comes from the Health Qigong Management Center of General Administration of Sport of China: Health Qigong-Baduanjin (2003).^[32]^ The whole exercise of Baduanjin 26 consists of 10 movements (including the preparatory movement and the finishing movement) (Fig. 3).

### 2.13 Control group

Participants in the control group did not receive any special sports training. These participants will be required to maintain their original lifestyle.

### 2.14 Follow up

After 24 weeks, all participants were expected to enter another 4-week follow-up period. At a later stage, participants will return to their original lifestyle. The research assistant will conduct a telephone follow-up or home visit every Monday morning. Information about participants’ subjective feelings, medication, and daily activities will be recorded. Primary and secondary outcomes will be assessed at the end of the follow-up period.

### 2.15 Participant retention and persistence

The success of the intervention depends to a large extent on the dynamic participation of participants. In order to stimulate the active participation of participants, the staff will adopt several strategies to improve the compliance of the intervention plan:

(1) After the participants are randomly assigned to the intervention group, the researcher will elaborate on the benefits of the Baduanjin;

(2) Professional teachers will guide the patients to carry out Baduanjin training, which will stimulate the participation of patients;

(3) The research assistant reminded the subjects to carry out the Baduanjin exercise according to the wechat group's learning plan and complete the Baduanjin training under the supervision of professional teachers.

The attendance rate of Baduanjin training will be evaluated by recording the training days. Patients who do not attend the training course will be considered as absenteeism.

In order to improve the retention rate of participants, subjects will be randomly assigned, and the research assistant will make every effort to ensure that the subjects remain normal throughout the study period.

It includes the following ways:

(1) the assistant researcher will maintain the participation of participants through interviews and telephone calls;
(2) Communicate regularly through materials, dialogues, and other means to inform the participants of our support;
(3) Flexible arrangement of learning time and the resolution of a time conflict.

### 2.16 Outcome indicators

#### 2.16.1 Primary endpoint

The main outcome of our study will be the change in VAS score of pain from baseline to the completion of the intervention (week 24).^[30]^ Patients will quantify pain intensity by marking the most relevant points anchored by 2 descriptors on a 100 mm horizontal line: 100 for ”most imaginable pain” and 0 for ”no pain”.

#### 2.16.2 Secondary ending

Pain-related disability index is used to measure general disability related to pain.

The Pain Disability Index is a widely used and researched tool for measuring pain-related disability, especially in chronic low back pain, fibromyalgia, cancer, or chronic generalized pain.^[33]^ Pain Disability Index is mainly composed of 7 projects, including family responsibilities, entertainment, social activities, occupation, sexual behavior, self-care, and life support activities. Each item is evaluated using a numerical score scale of 0 to 10, where 0 means no disability, 10 means the highest disability, and the total score is 70 points. The higher the score, the more the pain interferes with daily activities. Pain Disability Index is easy to understand and is an effective and reliable tool.

The Quebec back pain disability scale is used to measure disability related to specific back pain.^[34]^

The Quebec back pain disability scale is mainly used to assess the difficulty of various daily activities patients with low back pain. These activities are mainly divided into 6 areas, across a total of 20 questions: bed rest (question 1–3), sitting (question 4–6), walking (question 7–9), exercise (question 10–12), bending over (question 13–16) and carrying heavy objects (questions 17–20). Each question has 6 options, with a score of 0 to 5. A score of 0 means no difficulty, a score of 5 means unable to complete, and a higher score means more severe dysfunction. The total score is 100 points.

Measure the health-related quality of life through the SF-36 Health Survey.^[35]^

Health-related quality of life is evaluated using SF-36, which is a routine health questionnaire. The scale has 36 items divided into 8 dimensions: physical function, physical function, physical pain, general health, energy, social function, emotional function, and mental health. The first 4 dimensions belong to the field of physiology, while the last 4 dimensions belong to the field of psychology. The conversion ratio score of each field and each dimension of SF-36 is 0–100 minutes; the higher the score, the better the Health-related quality of life.

Roland Morris Disability Questionnaire (RMDQ), which has 24 items, from the affect overview disease (Sickness Impact Profile) simplifies RMDQ.^[36]^ It chooses 24 foods closely related to lower back pain. RMDQ is used to assess the condition of patients with low back pain within 24 hours before the test. These include walking, standing, bending, bed rest, clothes, sleep, self-care, and daily activities such as 8 aspects. The answer to each question is “yes” (1 point) or “no” (0 points). The total score is 0 to 24 points; the higher the score, the more severe the dysfunction. Some researchers believe that RMDQ has good reliability and effectiveness in assessing short-term changes before and after treatment for low back pain.

Global Perceived Effect is used (-5 to +5) to measure the overall perceived therapeutic effect.^[37]^

Overall Perceived Effect: This is the patient's subjective perception of the degree of pain, which is more used for the patient's perception of pain changes. Overall Perceived Effect is a 7-point system with the following specific regulations: 1 point: more damage than before; 2 points, more painful than before; 3 points, a little less damage than before; 4 points, no change; 5 points, pain is slightly improved; 6 points, pain improvement is more obvious; 7 points, almost no pain.

### 2.17 Economic analysis

Cost-effectiveness studies will be conducted simultaneously. Cost data will be collected using a separate economic analysis case report form. Medical expenses will include official medical expenses (paid by patients to the hospital) and unofficial medical expenses, including non-prescription drugs, health food, medical equipment and other required expenses. Non-medical costs include transportation costs, care costs and time costs. Loss of work efficiency will also be assessed.

### 2.18 Assess safety

Each examination will be a physical examination, and vital signs and adverse events will be checked. To check the safety of Baduanjin, blood pressure, heart rate, blood tests and nervous system checks will be performed during the first and fourth data collection. Any adverse events during the clinical trial will be documented and evaluated in the case report form. The incidence of adverse events will be calculated. Adverse events associated with the intervention will be monitored and appropriately addressed.

### 2.19 Demographic measures

Demographic data table, including; age, gender, height, weight, race, religion, marital status, smoking status, previous back pain episodes, previous back pain treatment methods, current treatment methods, and current exercise activities, will be completed during the assessment.

### 2.20 Data management and quality control

The study used paper case report form to collect data that had been approved by the Ethics committee. All the original paper data will be managed by specialized personnel to ensure the safety of patient data. At the end of the study, all documents will be safely stored for 5 years. All practitioners and researchers have been trained in good clinical practice. In order to improve the quality of Baduanjin, patients will be trained by professional teachers with more than 5 years of experience.

### 2.21 Statistics

All data are statistically analyzed using Statistical Product and Service Solutions (SPSS©), version 20.0 (IBM Corp., Armonk, NY, USA). Continuous variables will be mapped by the mean and its SE (normal distribution) or median and IQR (non-normal distribution). Categorical variables will be delineated as a frequency or percentage. The baseline characteristics between the comparison groups will be compared using the *t* -test or Mann-Whitney *U* test for continuous variables, while Pearson χ^2^ or Fisher exact test will be employed to compare categorical variables. The analysis of primary or secondary results will be established with the intent to treat principle, and missing data will be interpolated using multiple imputation methods. Student's *t* test or Mann-Whitney *U* test will be used to analyze the difference between the intervention group and the control group at each time period (8 weeks after the intervention, 16 weeks, and 24 weeks or 4 weeks of follow-up). In the safety analysis, the total adverse event rate and adverse event rate associated with Baduanjin were tested by Pearson χ^2^ or Fisher's exact test.

Primary analysis: treatment efficacy. The statistical analysis of primary and secondary results will be based on treatment principles. The effect of the intervention on pain distress, pain intensity, function, disability, and overall sensory effects will be determined using a linear mixed model (random intercept and fixed coefficient), which includes treatment, time, and time interaction. The treatment coefficient of time interaction provides an estimate of the effect of Baduanjin intervention. The magnitude of the treatment effect will be calculated for each follow-up time point. If there is a statistically significant treatment effect at any point in time, we will also calculate the number of treatments (NNT) required to achieve pain recovery.

### 2.22 Ethics

The research protocol complies with the Declaration of Helsinki. The ethics approval letter and consent letter of the research protocol came from the ethics committee of Nanping first hospital (approval number: NPSY202005001). The research background and main objectives, as well as potential benefits and risks, will be fully explained to participants and their families. Before participating in the study, participants will sign an informed consent form.

### 2.23 Dissemination

The research protocol has been registered and can be used on the website of the Chinese Clinical Trial Registration Center (http://www.chictr.org.cn/, with the identifier ChiCTR2000033908). The research results will first be communicated to each participant, and then disseminated to researchers, healthcare providers, healthcare professionals, and the public through courses, presentations, and the Internet, regardless of the degree or direction of its impact. The results will also be recorded in a peer-reviewed academic journal in the publication.

## 3 Discussion

In our study, after 24 weeks of training on Baduanjin and 4 weeks of follow-up. To observe the effect of Baduanjin on the main symptoms of chronic low back pain from the perspective of pain and dysfunction. In terms of quality of life, degree of disability and overall perception, to verify the impact of Baduanjin on patients’ daily life quality. Our research also provides safety assessment and economic benefit analysis. Baduanjin is a traditional Chinese exercise method. In recent years, more and more studies have used Baduanjin to assist the treatment of chronic obstructive pneumonia, coronary heart disease, hypertension and other diseases, as well as the rehabilitation of spinal joint diseases.^[38–42]^ Some previous studies have also confirmed the beneficial effects of Baduanjin on CLBP in terms of symptoms and activities.^[23]^ Some opinions believe that Baduanjin mainly uses the spine and waist as the axis to exercise, which is conducive to improving waist activity, improving waist muscle strength, and enhancing waist metabolism.^[43]^ Baduanjin requires patients to adjust their breathing and enter meditation.^[25]^ The state is conducive to the relaxation of tense muscles and the relief of pain. Nevertheless, the current design of the safety and effectiveness of Baduanjin in the treatment of chronic low back pain is still lacking. Therefore, this study will provide evidence on the usefulness of Baduanjin to the patient population.

Our research has many advantages. First of all, All Baduanjin are personally guided by professional teachers of more than 5 years to improve the quality of experimental data. Second, we will not only assess pain intensity, but also clinical relevance, quality of life, level of disability, and overall assessment. Third, the intervention effect of each recorded time point was analyzed statistically, build a hybrid linear model, to provide basic data for the treatment course and training frequency of Baduanjin. Fourth, economic evaluation is included for the first time to provide data for the urban benefit of Baduanjin. Fifth, safety index evaluation is included for the first time to verify the safety of Baduanjin training. However, our research also has some limitations. First of all, our sample size calculation may be overestimated. According to previous study, the duration of treatment was only 8 weeks, while ours was 24 weeks. Secondly, the control group used in the study is different from ours, which may affect the results of the study. Finally, the daily activity of the patients in the control group could not be quantified. After the inclusion of the patients, we could only see those with significantly increased or decreased daily activity, and carried out elimination management, but we could not make quantitative comparison with the patients in the intervention group.

Despite its limitations, the results of this trial are expected to provide evidence for the efficacy, safety and economic benefits of Baduanjin in the treatment of chronic low back pain. To provide medical staff, medical staff and patients with long-term exercise therapy plan recommendations.

## Author contributions

XMC and QTY conceived and designed this study. QTY and SLY drafted and revised the manuscript and will also perform data analysis for the primary and secondary outcome. JBW will conduct statistical analysis on other results in this trial. CYZ and XFL were responsible for cleaning up data and will participate in data analysis. DBY was responsible for query resolution and the integrity of the data. XMC supervised the project. All authors read and approved the final manuscript.

**Conceptualization:** Qingtang Yang, Xiangmei Chen.

**Data curation:** Caiyun Zheng, Xiaofeng Liang, Debiao Yu.

**Formal analysis:** Qingtang Yang, Jianbin Wang, Caiyun Zheng, Xiaofeng Liang.

**Investigation:** Debiao Yu.

**Supervision:** Xiangmei Chen.

**Writing – original draft:** Qingtang Yang, Shiliang Yu.

**Writing – review & editing:** Qingtang Yang, Shiliang Yu.

## Abbreviations

Abbreviations: CLBP = Chronic low back pain, RMDQ = Roland Morris Disability Questionnaire, VAS = Visual Analog Scale.
